# Predominant role of gut-vagus-brain neuronal pathway in postoperative nausea and vomiting: evidence from an observational cohort study

**DOI:** 10.1186/s12871-021-01449-9

**Published:** 2021-09-29

**Authors:** Nana Li, Lu Liu, Menghan Sun, Ruiliang Wang, Wenjie Jin, Cunming Liu, Youli Hu

**Affiliations:** 1 Department of Anesthesiology, The First Affiliated Hospital of Nanjing Medical University, Nanjing, 210029 P.R. China; 2 Department of Anesthesiology, Wuxi Second Hospital Affiliated Nanjing Medical University, Wuxi, 214002 P.R. China; 3 Department of Anesthesiology, Zhongda Hospital, Medical School, Southeast University, Nanjing, 210029 P.R. China

**Keywords:** Postoperative nausea and vomiting, Vagotomy, Emetic neuronal pathway

## Abstract

**Background:**

Postoperative nausea and vomiting (PONV) as a clinically most common postoperative complication requires multimodal antiemetic medications targeting at a wide range of neurotransmitter pathways. Lacking of neurobiological mechanism makes this ‘big little problem’ still unresolved. We aim to investigate whether gut-vagus-brain reflex generally considered as one of four typical emetic neuronal pathways might be the primary mediator of PONV.

**Methods:**

Three thousand two hundred twenty-three patients who underwent vagus nerve trunk resection (esophagectomy and gastrectomy) and non-vagotomy surgery (hepatectomy, pulmonary lobectomy and colorectomy) from December 2016 to January 2019 were enrolled. Thirty cases of gastrectomy with selective resection on the gastric branch of vagus nerve were also recruited. Nausea and intensity of vomiting was recorded within 24 h after the operation.

**Results:**

PONV occurred in 11.9% of 1187 patients who underwent vagus nerve trunk resection and 28.7% of 2036 non-vagotomy patients respectively. Propensity score matching showed that vagotomy surgeries accounted for 19.9% of the whole PONV incidence, much less than that observed in the non-PONV group (35.1%, *P* <  0.01). Multivariate logistic regression result revealed that vagotomy was one of underlying factor that significantly involved in PONV (OR = 0.302, 95% CI, 0.237-0.386). Nausea was reported in 5.9% ~ 8.6% vagotomy and 12 ~ 17% non-vagotomy patients. Most vomiting were mild, being approximately 3% in vagotomy and 8 ~ 13% in non-vagotomy patients, while sever vomiting was much less experienced. Furthermore, lower PONV occurrence (10%) was also observed in gastrectomy undergoing selective vagotomy.

**Conclusion:**

Patients undergoing surgeries with vagotomy developed less PONV, suggesting that vagus nerve dependent gut-brain signaling might mainly contribute to PONV.

## Introduction

Postoperative nausea and vomiting (PONV) is one of the most common postoperative complications. Inhalational agents, opioid analgesic, and several types of surgeries (cholecystectomy, gynecological, and laparoscopic surgery) are closely associated with increased risk of PONV [1–9]. With effective prophylaxis by using anti-emetic agents to block the activation of a wide range of neurotransmitter pathways via serotonin 5-HT3, dopamine D2, and histamine H1, PONV prevalence is still seen in approximately 30% patients with the recognized risk factors (female sex, smoking status, history of PONV or motion sickness and expected postoperative opioids [10, 11]. Because it cannot be predicted which neuronal pathway is postoperatively involved, multimodal antiemetic medications targeting at different mechanisms of action is inevitably used to the patients either at high risk of PONV or require rescue anti-emetics.

The vagus nerve is an important neuronal component of gut-vagus-brain pathway. Through mediating the bidirectional communication of the gut-brain axis, it regulates the ingestive behavior and even nausea and vomiting [12]. There are four neural pathways potentially responsible for triggering vomiting by direct projections to the nucleus of the solitary tract (NTS) in the hindbrain: 1) gut vagal afferent fibers innervating the stomach and intestine which are stimulated by paracrine factors (e.g., serotonin) [10, 13–15]; 2) motion-related vestibular input from the vestibule in the inner ear [10, 16, 17]; 3) area postrema (AP) potentially detects circulating toxins [18] and 4) descending pathways from the forebrain [19, 20]. Vagotomy and AP ablation in dog and ferret revealed that opioids produce emesis by action on the AP [21] while isoflurane-induced emesis is mediated by an action on the hindbrain rather than the abdominal vagus [22]. It is therefore argued that PONV might be the potential result of anesthetic agents performing their primary actions either in the gut or the brain. However, substantial evidence as to which one of these four neuronal pathways is the primary mediator of PONV is still lacking.

To clarify the potential role of intact gastric-vagal-brain reflex in the occurrence of PONV, we performed the cohort study in patients undergoing foregut surgeries including esophagectomy with the reconstructed gastric tube (vagotomy involving vagus nerve trunk resection), gastrectomy with gastrojejunostomy with subdiaphragmatic vagotomy, and other three non-vagotomy surgeries including hepatectomy, pulmonary lobectomy, and hindgut surgery (colorectomy). Furthermore, we further observed PONV incidence after gastrectomy with selective removal of gastric branch of vagus nerve.

## Methods and materials

The study was designed as an observational cohort study to investigate the difference of PONV incidence as the result of vagotomy with vagus nerve trunk resection. We collected data from five types of surgical procedures which were further divided into two groups: vagotomy (esophagectomy and gastrectomy) and non-vagotomy (hepatectomy, pulmonary lobectomy and colorectomy) groups. Furthermore, 30 cases of gastrectomy undergoing selective resection on the gastric branch of vagus nerve were also recruited in this study. The study was approved by the Ethics Committee of the First Affiliated Hospital of Nanjing Medical University (protocol number SR-396).

In-patients scheduled to undergo elective surgery at the First Affiliated Hospital of Nanjing Medical University from December 2016 to January 2019 were enrolled in this study. They underwent one of five procedure types requiring general anaesthesia and follow-up for the first post-operative day. Patients were excluded from this study according to the exclusion criteria: (1) less than one-hour operation time; (2) over 800 ml intraoperative blood loss; (3) incomplete data. The premdication, anaesthetic techniques and postanaesthetic care were performed by experienced anaesthetic staff based on their usual practice.

### Perioperative medication management

Routinely, intravenous infusion of propofol and remifentanil along with 1% sevoflurane were used for the maintenance of anesthesia. Fentanyl at 10 μg/kg was applied for the patients during surgery, while the opioid analgesics for postoperative patient-controlled analgesia was 0.15 mg/kg butorphanol±0.5 mg fentanyl. The perioperative anti-emetic paradigm included: 1) 10 mg dexamethasone prior to the anesthesia induction; 2) intra-operative 5-HT3 antagonist (granisetron); 3) postoperative 9 mg granisetron along with patient-controlled opioid analgesia. Laparoscopy was performed in gastrectomy, colorectomy and pulmonary lobectomy, while most of hepatectomy and esophagectomy were undergoing laparotomy.

### Data collection

The questionnaire contained items including patient characteristics as well as details of anaesthesia and surgery performed and items on the postoperative outcome under observation. The anaesthetic nurses completed the items on the surgical procedure, the premedication and the anaesthetic given based on the data extracted from local hospital information system. The follow-up interview team interviewed patients with focus on the items covering postoperative nausea and vomiting and its treatment, medication for postoperative pain on the first day after surgery. The assessment intervals were the initial observation in the recovery room and the following interview on the surgical ward at 24 h after the operation. The interviewer firstly recorded emetic episodes, anti-emetics used and pain medication from the medical notes before visiting the patient. The patients were then interviewed with the questionnaire about the presence of nausea, intensity of vomiting and pain postoperatively and the overall satisfaction with surgery, anesthesia and postoperative care.

### Assessment of symptoms and outcome

For the aim of this study, we were interested in the effect of vagus nerve truck resection on the incidence of PONV. The primary outcome of the study was the incidence of PONV which was defined as occurrence of nausea and vomiting within 24 h after the surgery. Nausea was evaluated by the patient’s subjective sensation of feeling sick or wishing to vomit without further intensity assessment. Emetic episodes were recorded as both retching and vomiting since occurrence of sole retching was relatively rare in this study. The intensity of vomiting was graded into two levels: mild (< 3 times) and severe (≥3 times). Patients experienced retching only were recorded as mild vomiting.

### Covariates

Previous studies and guidelines showed that the risk factors of PONV were female gender, nonsmoking status, history of motion sickness, age, the use of inhaled anesthetics, postoperative opioid, in addition to ASA physical and duration of anesthesia [23, 24]. All these data except smoking status and history of motion sickness were collected as covariates.

### Statistical analysis

Numbers and percentages were used to represent categorical variables and continuous variables by mean values with standard deviation (SD), t-test, and x^2^ test was used to compare continuous variables and categorical variables between groups. Variables were compared between different groups by using univariate analysis. Variables with statistically significant differences in univariate analysis were entered into a multivariate model by the forward condition method.

Next, we performed propensity score matching analysis to reduce bias by using the nearest neighbor method and one-to-one matching with caliper set at 0.1. Variables used for matching were gender, age, BMI, inhaled anesthesia, intraoperative fentanyl usage, postoperative opioid (patient-controlled analgesia), ASA physical, duration of anesthesia, blood loss, fluid infusion, laparoscopic procedures and vagus nerve trunk resection. All statistical analyses were carried out in SPSS 22.0 (SPSS Inc., Chicago, IL USA), and *P* <  0.05 was considered statistically significant.

## Result

### Patient characteristics

**Fig. 1:**
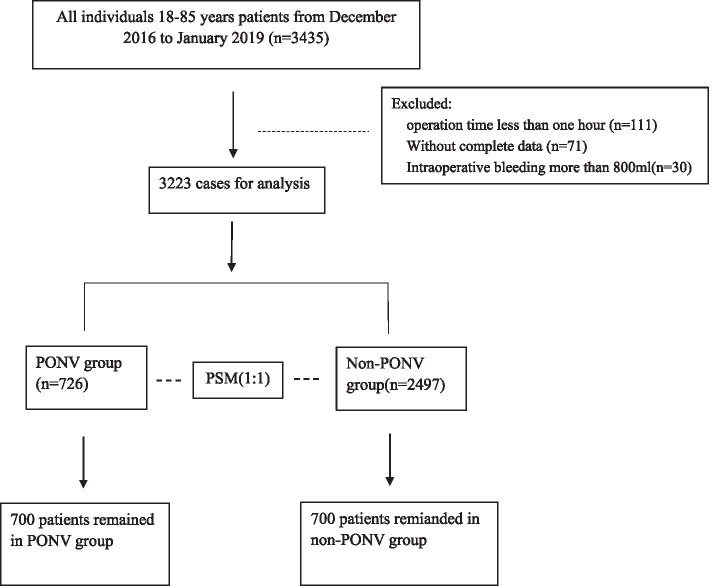
A flowchart elucidating the patients selection process and grouping methods included in this observational cohort study

There were total 3435 patients who underwent five types of surgical procedures questionnaired and interviewed, with 3223 patients remaining in this study after exclusion criteria applied as operation time less than 1 h (*n* = 111), incomplete data (*n* = 71) and intraoperative bleeding more than 800 ml (*n* = 30)) (Fig. 1). They are initially divided into PONV (*n* = 726) and non-PONV (*n* = 2497) groups. To reduce bias, a matched patient group was established for propensity score matching analysis between the two groups (*n* = 700 equally in each group).

The demographic characteristics of the patients in PONV and non-PONV groups were showed in Table 1. 31.2% of non-PONV incidence occurred in female patients, while male patients were much less likely to have nausea and vomiting after surgery as compared with females (38.8% vs 61.2%). The operation time was slightly shorter in the PONV group. Intraoperative crystal/colloid fluid infusion and fentanyl consumption were slightly more applied in the non-PONV group. Other characteristics including ASA physical status, age, BMI, blood loss and laparoscopic procedures were quite similar in both groups. In the whole PONV incidence, use of inhaled anesthetics and postoperative opioid (patient-controlled analgesia) account for 83.7 and 82.6% respectively.

**Table 1:** Comparison of patient characteristics data and prospensity score matching analysis between non-PONV and PONV group in observational cohort study

Variables	Before propensity score matching		X^2^/t	*P*-Value	After propensity score matching		X^2^/t	*P*-Value
	non-PONV group (*n* = 2497)	PONV group (*n* = 726)			non-PONV group (*n* = 700)	PONV group (*n* = 700)		
Female (n,%)	778 (31.2%)	444 (61.2%)	215.045	< 0.001	434 (62.0%)	418 (59.7%)	0.768	0.381
Age (year)	60.0 ± 11.1	59.1 ± 11.1	− 1.914	0.056	58.4 ± 12.0	58.9 ± 10.9	0.803	0.420
Age (year)			4.495	0.034			0.011	0.915
≦ 60	1144 (45.8%)	365 (50.3%)			355 (50.7%)	353 (50.4%)		
> 60	1353 (54.2%)	361 (49.7%)			345 (49.3%)	347 (49.6%)		
BMI	23.5 ± 3.1	23.3 ± 3.2	−2.056	0.040	23.5 ± 3.2	23.3 ± 3.1	−1.348	0.178
ASA physical status			2.188	0.534			0.255	0.880
I	172 (6.9%)	51 (7%)			56 (8.0%)	51 (7.3%)		
II	2063 (82.6%)	608 (83.8%)			581 (83.0%)	585 (83.6%)		
≥ III	262 (10.5%)	67 (9.2%)			63 (9. 0%)	64 (9.1%)		
Duration of anesthesia (h)	2.6 ± 1.0	2.3 ± 1.0	−5.243	< 0.001	2.3 ± 0.9	2.3 ± 1.0	0.551	0.582
Laparoscopic surgery	1730 (69.3%)	528 (72.7%)	3.181	0.075	504 (72.0%)	502 (71.7%)	0.014	0.905
Crystalloid solution (ml)	1305.3 ± 462.1	1238.7 ± 417.7	−3.491	< 0.001	1225.7 ± 436.5	1242.1 ± 418.5	0.718	0.420
Colloid solution (ml)	614.1 ± 318.0	572.2 ± 303.9	−3.309	0.001	562.1 ± 282.9	571.4 ± 307.2	0.590	0.555
Bood loss (ml)	147.7 ± 190.7	134.6 ± 173.7	−1.661	0.097	131.3 ± 168.2	134.9 ± 174.3	0.399	0.690
Intraoperative fentanyl usage (mg)	0.5 ± 0.2	0.5 ± 0.1	−3.965	< 0.001	0.5 ± 0.1	0.5 ± 0.1	0.628	0.530
Postoperative opioid (patient-controlled analgesia)	2360 (94.5%)	600 (82.6%)	105.726	< 0.001	610 (87.1%)	600 (85.7%)	0.609	0.435
Inhaled anesthesia	1633 (65.4%)	608 (83.7%)	89.381	< 0.001	569 (81.3%)	582 (83.1%)	0.826	0.364
Vagus nerve trunk resection	1046 (41.9%)	141 (19.4%)	122.052	< 0.001	246 (35.1%)	139 (19.9%)	41.017	< 0.001

Notably, as compared with non-vagotomy surgeries (hepatectomy, pulmonary lobectomy and colorectomy), vagus nerve trunk resection performed in both esophagectomy and gastrectomy significantly reduced PONV incidence by approximately 4-fold, dropping from 80.6 to 19.4%. This result suggested that vagus nerve stimulation might play a predominant role in triggering PONV occurrence.

### Vagotomy associated with PONV in the multivariate logistic regression model

To further examine the potential role of vagus nerve in PONV, we performed multivariate logistic regression analysis with factors associated with PONV in entire cohort (Table 2). There are four variables examined including gender, inhaled anesthetics, postoperative opioid and vagotomy. As expected, gender is one of risk factors of PONV as male patients had an OR value of 0.306 (95% CI, 0.253-0.369) as compared with females. In addition, the use of inhaled anesthetics was highly involved in PONV (OR = 4.020, 95% CI, 3.189-5.067). Much lower incidence of PONV (OR = 0.326, 95% CI, 0.245-0.433) with postoperative opioid application was unexpectedly found in multivariate logistic regression analysis, in contrast to common point of view which consider postoperative opioid as a risk factor for PONV. This might be duo to: 1) dexamethasone being regularly used in premedication along with intra-operative 5-HT3 antagonists; 2) sufficient anti-emetic (normally 9 mg granisetron) accompanying opioid analgetics (butorphanol±fentanyl at much lower dosage) during postoperative patient-controlled analgesia in our hospital; 3) butorphanol is less likely to cause PONV [25].

**Table 2:** Multivariate logistic regression analysis with factors associated with PONV in observational cohort study

Variables	OR (odds ratio)	95% CI	*P*-value
Sex (male vs female)	0.306	0.253 ~ 0.369	< 0.001
Inhaled anesthesia	4.020	3.189 ~ 5.067	< 0.001
Postoperative opioid (patient-controlled analgesia)	0.326	0.245 ~ 0.433	< 0.001
Vagus nerve trunk resection	0.302	0.237 ~ 0.386	< 0.001

*CI* Confidence interval

Multivariate logistic regression result revealed that vagus nerve trunk resection served as one factor that significantly modulated the occurrence of PONV (OR = 0.302, 95% CI, 0.237-0.386), further providing the evidence that vagus nerve works as a primary afferent nerve to receive PONV-related signal inputs. These data also support the potential underlying mechanism by which intact vagus nerve might play a key role for PONV.

### Vagus nerve trunk resection is associated with lower incidence of PONV

To further investigate the role of vagus nerve in PONV, we performed propensity score matching to adjust the imbalance of concomitant variable in order to avoid the bias (Table 1). Propensity score matching is an effective method to analyze the observational data when randomized trials are not feasible [26]. After propensity score matching, 700 patients remained in each PONV and the non-PONV group. Gender, age, BMI, the use of volatile anesthetics, postoperative opioid, ASA physical, duration of anesthesia, laparoscopic surgery, intraoperative fluid infusion, blood loss and fentanyl usage were similar between two groups. Notably, 19.9% of PONV and much more (35.1%, *P* <  0.001) non-PONV cases were seen in patients undergoing surgeries (esophagectomy and gastrectomy) with vagus nerve trunk being resected. Consistently, more PONV incidence (80.1%) was observed in patients undergoing surgery with intact vagus nerve (hepatectomy, pulmonary lobectomy and colorectomy). This result also suggests that vagus nerve trunk resection is associated with the incidence of PONV.

### Emetic outcomes of vagotomy and non-vagotomy

As crucial role of vagus nerve trunk resection was confirmed through multiple statistical analysis, we further evaluated the detailed percentage of nausea and the intensity of vomiting (Table 3). Most of patients with esophagectomy and gastrectomy in vagus nerve trunk resection group did not report nausea and vomiting (90.9 and 86.5% respectively), while the percentage of non-PONV was reduced to approximately 70% in patients without vagus nerve trunk resection. The overall incidence of PONV were much lower in vagotomy patients with 9.1% in esophagectomy and 13.5% in gastrectomy. By contrast, when intact vagus nerve was maintained, the highest incidence of PONV (~ 30%) was reported in pulmonary lobectomy and hepatectomy, followed by 23% of PONV incidence in colorectomy patients. Consistently, nausea was reported in 5.9% esophagectomy patients and 8.6% gastrectomy patients as compared with that in 12 ~ 17% patients without vagotomy. Most vomiting episodes were less than 3 times, being around 3% in vagotomy patients including esophagectomy and gastrectomy and 8 ~ 13% in non-vagotomy patients. Sever vomiting graded as more than 3 times were much less experienced in patients undergoing vagotomy (~ 1%) or non-vagotomy (~ 3%).

**Table 3:** Details of PONV between two groups with and without vagus nerve resection including nerve trunk resection and selective vagotomy

	Non-PONV	PONV			
		nausea	Vomiting < 3 times	Vomiting >3times	total
Vagus nerve trunk resection group (n,%)					
Esophagectomy (*n* = 441)	401 (90.9%)	26 (5.9%)	10 (2.3%)	4 (0.9%)	40 (9.1%)
Gastrectomy (*n* = 746)	645 (86.5%)	64 (8.6%)	29 (3.9%)	8 (1.1%)	101 (13.5%)
Total (*n* = 1187)	1046 (88.1%)	90 (7.6%)	39 (3.3%)	12 (1.0%)	141 (11.9%)
Gastrectomy with selective vagotomy (*n* = 30)	27 (90.0%)	2 (6.6%)	1 (3.3%)	0 (0.0%)	3 (10.0%)
Non-vagus nerve trunk resection group (n,%)					
Colorectomy (*n* = 574)	442 (77.0%)	71 (12.4%)	46 (8.0%)	15 (2.6%)	132 (23%)
Hepatectomy (*n* = 524)	366 (69.8%)	87 (16.6%)	57 (10.9%)	14 (2.7%)	158 (30.2%)
Pulmonary lobectomy (*n* = 938)	643 (68.6%)	144 (15.4%)	122 (13.0%)	29 (3.1%)	295 (31.4%)
Total (*n* = 2036)	1451 (71.3%)	302 (14.8%)	225 (11.1%)	58 (2.8%)	585 (28.7%)

### Selective vagotomy and specific role of gastric-vagal-brain reflex in PONV

To further investigate the role of specific branches of vagus nerve involved in PONV, we analyzed 30 cases of gastrectomy undergoing selective vagotomy by specifically dissecting gastric branches of vagus nerve (Table 3). 90% of gastrectomy patients with selective vagotomy did not experience PONV, while only 10% patients presented PONV (6.6% for nausea and 3.3% for mild vomiting). The PONV incidence following removal of gastric branches of vagus nerve was quite similar to that with vagus nerve trunk resection, further supporting the crucial role of gastric-vagal-brain reflex in the neurobiological mechanism underlying PONV.

## Discussion

**Fig. 2:**
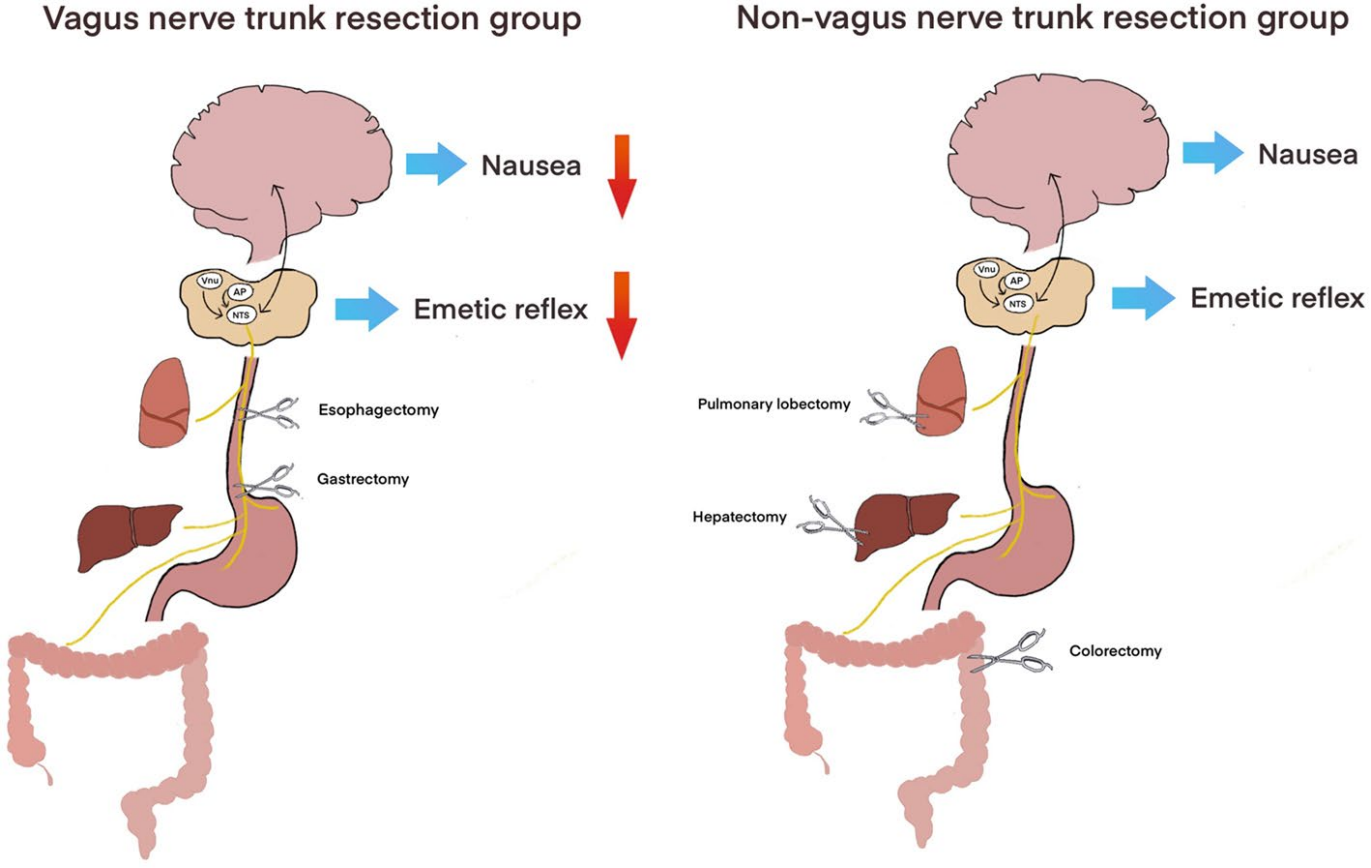
Schematic diagram illustrating the effect of vagotomy on PONV. Four neural pathways potentially send stimulating inputs to the nucleus of the solitary tract (NTS) in the hindbrain: 1) gut vagal afferent fibers (yellow line) from the gastrointestinal tract; 2) motion-related vestibular input from the vestibular nuclei (Vnu); 3) area postrema (AP) and 4) descending pathways from the forebrain. NTS then produces the emetic reflex by activating its output pathways within local brainstem areas and causes nausea by projecting to the mid- and forebrain. However, which one of these four neuronal pathways as the primary mediator of PONV is still unknown. In this cohort study, occurrence of PONV is about 30% after non-vagotomy surgery (hepatectomy, pulmonary lobectomy and colorectomy), while PONV is reduced to approximately10% after vagotomy surgery (esophagectomy and gastrectomy) and selective vagotomy, suggesting that vagus nerve dependent gut-brain signaling mainly contributes to PONV

In the present study we found that based on the surgical procedures, vagus nerve trunk resection performed during esophagectomy and gastrectomy caused much less incidence of PONV as compared with that when vagus nerve trunk was intactly reserved (Fig. 2). PONV occurred in an increasing order as: esophagectomy < gastrectomy < colorectomy < hepatectomy and pulmonary lobectomy. As compared to the similar occurrence of PONV (23 ~ 30%) among the last three types of surgery, subdiaphragmatic vagotomy and inherent trunk vagotomy commonly seen in gastrectomy and esophagectomy reduced PONV to the lowest extent at 9.1 and 13.5% respectively. These observations indicated that stomach and esophagus with intact vagal nerve innervation should work as primary site to trigger PONV, as vagotomy accounts for approximately three-fold decrease on PONV. This is further supported by the fact that selective vagotomy with gastric branch of vagus nerve resection kept the PONV incidence at the lowest level. Hence, the hindgut, liver and lung surgery could create PONV-relevant neuronal and/or endocrine signals to activate the common afferent neuronal pathways typically via the gastrointestinal tract. In addition, the gastro-vagal independent neuronal circuits including AP, vestibular and forebrain pathways are much less involved in PONV.

The vagus mediates reciprocal communication between gut and brain. It is known that signals from the gut could make activation on the vagus through the enteric sensory system. Gastric dysrhythmias as an established biomarker for nausea and vomiting could stimulate the vagus following the stimulation on local enteric neurons [27, 28]. Therefore after some potentially PONV-inducing types of surgery including laparoscopic and gynecological surgery, common side-effects such as postoperative ileus and subsequent disruptions of gastrointestinal motor rhythms could contribute to the etiology of PONV [29]. Both opioids and inhalational anesthesia also disrupt gastrointestinal function. It is known that inhalational anesthetic agents (halothane, isoflurane, and sevoflurane) stimulate vagal afferent fibers in dogs [30]. Furthermore, surgical manipulation and trauma could produce local release of substance P, 5-HT, or other mediators that affect signaling of extrinsic afferent fibers [15, 31], while inhaled anesthetics can enhance 5-HT3 receptor function [32]. Given the high levels of serotonin in the gut, exposure of the gut to the surgical procedures and anesthetics might increase the excitability of intrinsic primary afferent neurons in the myenteric plexus and then vagal nerve, contributing to the PONV. Indeed, much less PONV observed after vagotomy in this study suggest that in human, major role of gut-vagal pathway is actually underlying anesthetics-inducing emesis.

It is known that oesophagus can regulate the gastric motility via intact brainstem vago-vagal circuits. The NTS, pars centralis, receives oesophageal afferent projections from the vagus nerve and sends axons to the dorsal motor nucleus of the vagus to mediate parasympathetic control over the stomach. There are two neuronal subpopulations in dorsal motor nucleus which may be either activated or inhibited by oesophageal distension [33]. Intravenous and inhaled anesthetic agents could decrease the esophageal sphincter tone, the phenomenon being considered as a sign to monitor the anesthesia depth [34]. Therefore, anesthesia-induced esophageal distension might disrupt gastric motor rhythms by functioning differently on the vagal inhibitory and excitatory pathways to induce PONV.

The NTS and specific nuclei in area of the reticular formation, including the respiratory nuclear groups, as important sites for generating emesis [20, 35–37]. In the present study postoperative vomiting still occurred in about 3% patients even with esophageal vagotomy. This finding suggests that in addition to the major vagal pathway, specific brainstem nucleis such as the respiratory nuclear groups work as a complementary neuronal pathway for emesis, which is responsible for the surgical and anesthetic stimulus.

Nausea can occur separately from vomiting. The role of vagal nerve in the stimulation of nausea could be speculated from the observation that esophageal vagotomy reduced nausea by 3-4 fold, especially in female patients. The dorsal pons (potentially the parabrachial nucleus), amgydala, and putamen are considered as a relay to transmit sensory input from the NTS between the emetic circuitry and forebrain, which is underlying the neurobiological mechanism of nausea [38]. Hence, the vagal nerve innervated on the esophagus play an important role in surgery and anesthesia induced nausea by activating NTS-nausea circuitry-forebrain pathway.

The limitation in the present study is that the surgical vagotomy performed disrupts both the sensory and motor components of the vagus, making it impossible to resolve the role of only the sensory input versus motor output. Additionally, the spinal afferents supplying the gastrointestinal system could have become sensitive to emetic stimulus after vagotomy, an effect that occurs in the context of chemotherapy-induced emesis [39]. Furthermore, due to missing recording of history of motion sickness in some of follow-up data collection, we did not include motion sickness as one of PONV variables in this study. Finally, since this cohort study is a retrospective investigation, even though the logistic regression analysis and propensity matching scoring methods were applied, some of non-included confounding factors due to the lack of observational indicators might affect the conclusion of our study. Further prospective studies are needed to verify the role of the vagus nerve in PONV.

Clinically vagal stimulation is an FDA approved treatment for intractable depression and also has been used in the treatment of refractory epilepsy. Our results reveal the predominant role of gastric vagus nerve in the PONV, therefore potentially promoting new approaches to prevent/treat such uncomfortable postoperative side effect by selectively modulating peripheral vagal sensitivity and activity. Our findings support the view that vagus nerve dependent gut-brain signaling mainly contributes to the effects of PONV and further highlight the feasible neuronal approaches that may not even require therapeutic agents to enter the circulation.

## Data Availability

The datasets used and analyzed during the current study are available from the corresponding author on reasonable request.

## Abbreviations

PONV: Postoperative nausea and vomiting
OR: Odds ratio
SD: Standard deviation
PSM: Propensity score matching
CI: Confidence interval
NTS: Nucleus of the solitary tract
AP: Area postrema
5-HT: 5-hydroxytryptamine
